# Associations between anxiety, depression, and personal mastery in community-dwelling older adults: a network-based analysis

**DOI:** 10.1186/s12888-024-05644-z

**Published:** 2024-03-07

**Authors:** Zekun Bian, Renyan Xu, Bin Shang, Fei Lv, Weiyi Sun, Qian Li, Yijing Gong, Caifeng Luo

**Affiliations:** 1 School of Medicine, Jiangsu University, Zhenjiang, China; 2 Department of Nursing, Jingjiang College, Jiangsu University, Zhenjiang, China

**Keywords:** Personal mastery, Anxiety, Depression, Older adults, Network analysis

## Abstract

**Background:**

In China, about 18.70% of the population aged 60 years and older are at risk of low personal mastery as well as anxiety and depression for a variety of reasons. The purpose of this study was to construct a symptom network model of the relationship between anxiety, depression, and personal mastery in community-dwelling older adults and to identify central and bridge symptoms in this network.

**Methods:**

Depression, anxiety, and personal mastery were measured using the Patient Health Questionnaire-9 (PHQ-9), Generalized Anxiety Disorder Scale (GAD-7), and Personal Mastery Scale (PMS), respectively. A total of 501 older adults in 16 communities in Changzhou and Zhenjiang, Jiangsu Province, China, were surveyed by using a combination of stratified sampling and convenience sampling methods. The R language was used to construct the network.

**Results:**

(1) The network structure of anxiety–depression–personal mastery was stable, with “Nervousness” (node GAD1, strength = 1.38), “Sad mood” (node PHQ2, strength = 1.22), " Inability to change” (node PMS2, strength = 1.01) and “Involuntarily” (node PMS3, strength = 0.95) as the central symptoms. (2) “Irritability” (node GAD6, bridge strength = 0.743), “Sad mood” (node PHQ2, bridge strength = 0.655), and “Trouble relaxing” (node GAD4, bridge strength = 0.550) were the bridge symptoms connecting anxiety, depressive symptoms, and personal mastery. (3) In the network comparison test (NCT), residence, somatic chronic comorbidity and gender had no significant effect on network structure.

**Conclusions:**

The construction of the anxiety–depression–personal mastery network structure opens up new possibilities for mechanisms of action and intervention formulation for psychological disorders in community-dwelling older adults. The identification of central symptoms (e.g., nervousness, sad mood, inability to change, involuntarily) and bridge symptoms (e.g., irritability, sad mood, trouble relaxing) in community-dwelling older adults with anxiety, depression, and low sense of mastery can provide a scientific basis for the development of precise interventions.

**Supplementary Information:**

The online version contains supplementary material available at 10.1186/s12888-024-05644-z.

## Introduction

The results of the seventh Chinese census show that the population aged 60 and above accounts for about 18.70% of the total population in China. According to the United Nations definition of a mildly aging society (10% or more of the total population is aged ≥ 60), China has already entered a mildly aging society and is about to enter a moderately aging society (20% or more of the total population is aged ≥ 60). Among them, Changzhou City and Zhenjiang City in Jiangsu Province, with 20.01% and 23.56% of the total population aged 60 and above, respectively, have entered the moderate aging, and the two cities have a large base of elderly people and are highly representative. As older people age, with the deterioration of multiple organ functions and stressful life events, mental health problems come to the fore, manifesting themselves in the inability of older people to express and control their emotions appropriately, and their lack of security and motivation. This can lead to serious problems such as mental illness, physical illness, and even to suicide. And, there are fewer studies on the mental health of the elderly. Therefore, it is necessary to pay attention to the mental health of the elderly population to help them have a healthy and happy later life. Among these mental health problems, depression and anxiety are the most common, with an incidence of 25.5% [1] and 9.9% [2], respectively.

Studies have shown that anxiety and depression are longitudinally associated bidirectional risk factors [3] and that they are also strongly correlated at the symptom level, with symptoms of anxiety and depression being predictive of each other, and the occurrence of symptoms of either psychological problem may also lead to an increased risk of symptoms of the other disorder [4–6]. Therefore, depression and anxiety disorders often interact with each other and appear as comorbidities [7]. The prevalence of comorbidity between anxiety and depression has been found to be between 60 and 80% [8]. The occurrence of comorbid anxiety and depression often leads to more severe consequences than anxiety or depression alone, not only leading to more severe psychological symptoms, higher risk of somatic chronic illness and more severe cognitive impairment in individuals, but also to a much higher risk of suicide [9–15].

Personal mastery, which refers to [16] an individual's beliefs about their ability to influence the course and outcome of meaningful life events, is an important psychological resource for individuals to manage their emotions and cope with stress. As an individual's primary control decreases in old age, so does the level of personal mastery. The effect of this is that older people tend to lose the confidence in life that they possessed in middle age and youth, and show a lack of motivation in coping with life events and an inability to withstand the negative emotions that can be associated with some stressful events, such as anxiety and depression. Therefore, personal mastery, as a positive psychological resource, is particularly important in this fragile life stage of the elderly. It has been found [17] that personal mastery can have a moderating effect on individuals' negative emotions and psychological disorders [18] and is a protective factor against anxiety and depression [19]. Personal mastery and anxiety and depression are closely related [20] and interact with each other with a significant negative correlation [21–23]. Studies have shown that lower levels of personal mastery usually lead to higher levels of anxiety and depression [24, 25], while higher personal mastery can reduce the incidence of negative emotions such as anxiety and depression [26]. Most of the previous research on anxiety–depression has been on the association between anxiety–depression disorders and other psychological problems.With the development of positive psychology, researchers have tried to use more positive psychological resource perspectives to try to explain and intervene in the mechanisms that generate these psychological problems of anxiety and depression. Among them, personal mastery is an important part of positive psychological resources, but there are still not enough articles studying the relationship between personal mastery and anxiety and depression. Moreover, most of the previous studies on anxiety, depression, and personal mastery relied on total scores to determine the level and condition of the respondents, which is a generally superficial form of assessment that may ignore the connection between each specific symptom, in the process losing a great deal of meaningful information. This can lead to overly broad interventions for anxiety, depression, and personal mastery in the absence of precise intervention targets focusing on specific important symptoms, wasting much time, money, and effort on separate interventions for each symptom while failing to produce effective intervention.

Network analysis is an emerging research method in the field of psychology. Differently from the traditional latent variable modeling method, network analysis uses relationships between variables that affect each other to explain psychological characteristics, providing a new perspective for the explanatory and mechanistic research of psychiatric problems that helps make up for the limitations of traditional modeling methods. In addition, network analysis uses symptoms as nodes and relationships between symptoms as edges and visualizes the inter-relationships among nodes in the network through nodes and edges to form a network. Network analysis has been used to analyze the inter-relationships between psychiatric symptoms.Two studies from Korea jointly showed that PHQ2 "Sad mood" and PHQ9 "Suicide" were the central symptoms of the depressive symptom network in Korean older adults with subjective memory complaints [27] and in Korean community-dwelling older adults [28]. In two Chinese studies, the central symptoms in the symptom network of a sample of older adults with major depression during the COVID-19 pandemic urban blockade [29] were PHQ6 "Guilty," PHQ2 "Sad mood," and PHQ4 "Energy"; in the anxiety and depression symptom network of functionally impaired older adults [30], the central symptoms were PHQ2 "Sad mood" and GAD2 "Uncontrollable worry ".

To our knowledge, no studies have yet constructed symptom networks of anxiety, depression, and personal mastery, and we have no way of knowing the mechanism of action of personal mastery on anxiety and depression; therefore, we lack an effective basis for precise interventions for psychiatric disorders. Therefore, in view of the use of network analysis in previous studies, this study attempts to use network analysis to construct a symptom network model of anxiety, depression, and personal mastery in community-dwelling elderly people, hoping to use personal mastery as an entry point to conduct a more in-depth study of the mechanisms of psychiatric disorders and identify the central symptoms and bridge symptoms in the network. By focusing on these important nodes, we hope to develop more scientific and feasible precise intervention programs for the psychiatric disorders of community-dwelling elderly with anxiety and depression and low personal mastery.

## Methods

### Study design

This study is a cross-sectional survey. From January 2023 to May 2023, a combination of stratified sampling and convenience sampling was used to randomly select 8 streets in Zhenjiang and Changzhou, Jiangsu Province, China, in proportion to the household registration population and 2 communities in each street for a total of 16 communities.The questionnaires were then administered to eligible elderly people in the selected communities according to the convenience sampling method. 524 questionnaires were distributed, and 501 valid questionnaires were collected, with a valid recovery rate of 95.6%. The requirements for the survey respondents were (i) age ≥ 60 years old; (ii) clear mind and ability to understand the survey content;and (iii) voluntary participation in this study. Exclusion criteria were (i) non-resident status in the local community (permanent residence < 6 months) and (ii) being unable to cooperate with the survey due to physical reasons (e.g. hearing problems). All participants signed an informed consent form prior to participation in this study. This study received ethical approval from Jiangsu University School of Medicine, approval number JSDX20230602001. This study was completed using a paper-based questionnaire-type offline survey. A total of four investigators carried out the survey after uniform standardized training, and one survey was conducted per subject. The investigators explained the purpose and significance of this study and the survey content to the respondents in detail and obtained informed consent to conduct the survey. The investigators used a face-to-face question-and-answer survey with the elderly in the community, and the survey results were completed by the investigators. During the survey, entries that were not understood by the respondents were explained and the community seniors were encouraged to answer truthfully. Furthermore, we did not deliberately guide the the community senior during the course of our investigation. Data entry was performed using EpiData3.1 software, and the process was performed in pairs to ensure consistent and accurate data entry.

## Measures

The Generalized Anxiety Disorder (GAD-7) [31] was first introduced into China by the scholar He Xiaoyan [32] for Chinese translation and reliability testing. At this time, Cronbach′s α coefficient of the Chinese version of the GAD-7 in general hospital outpatients was 0.898 [32]. The GAD-7 has 7 items, each reflecting a symptom of anxiety, and is scored on a four-point Likert scale of 0 to 3 (not at all = 0, a few days = 1, more than half the time = 2, and almost every day = 3) with a total score of 0 to 21, with a score of ≥ 5 indicating anxiety symptoms. The higher the score, the more severe the anxiety situation. The Cronbach′s α coefficient for the GAD-7 in this study was 0.89. The Patient Health Questionnaire-9 (PHQ-9) was localized for China and tested for reliability by scholar Xu Yong [33] in 2007, and the Cronbach′s α coefficient of the Chinese version of the PHQ-9 in the Chinese community elderly population was 0.833 [33]. The PHQ-9 has 9 items, each representing a symptom of depression, and is scored on a four-point Likert scale of 0 to 3 (not at all = 0, a few days = 1, more than half the time = 2, and almost every day = 3) with a total score of 0 to 27, with a score of ≥ 5 indicating depressive symptoms. The higher the score, the more severe the depressive condition. The Cronbach′s α coefficient for the PHQ-9 in this study was 0.868. The Personal Mastery Scale (PMS) [34] contains seven items scored on a five-point Likert scale (not at all = 1, not quite = 2, somewhat = 3, basically = 4, and very much = 5) with the first five items scored inversely and a total score of 7 to 35, with higher scores indicating higher levels of personal mastery. The Chinese version of this scale had a Cronbach′s α coefficient of 0.81 in cancer patients [20]. The Cronbach′s α coefficient in this study was 0.656.

### Statistical analysis

This study used SPSS 26.0 for descriptive analysis, using means and standard deviations to describe anxiety, depression, and personal mastery scores of older adults in communities. The network analyses were performed on R 4.3.0 software.

### Network estimation

In this study, we used the graphical Least Absolute Shrinkage and Selection Operator (gLASSO) [35] in the qgraph (version 1.9.4) program package with the Extended Bayesian Information Criterion (EBIC) [36] method for network estimation. This EBICglasso algorithm has better specificity and can exclude those connections that do not exist in the real network [36]. In this study, we used Spearman correlation analysis on the basis of this algorithm to further exclude indirect connections that existed between nodes.In the constructed network model, the thickness of the edges represents the weight of the edges, which indicates the closeness of the relationship between the nodes. The color of the edges indicates the nature of the relationship between the nodes (in this study, blue edges indicate positive relationships and red edges indicate negative relationships). We used the mgm (version 1.2–13) package to calculate the predictability of a node, which is the variance in a node that can be explained by all other nodes in the network.

### Network characterization

The centrality metrics of nodes [37], which are used to quantify the degree of importance of nodes in a network structure, include strength, tightness, and intermediation. Previous studies [38] have shown that tightness and intermediation are not stable enough to measure the importance of nodes, while strength can be a good indication of the centrality of nodes. Therefore, this study uses strength as a measure of node centrality. Strength is the sum of all connected edge weights of a node in a network [9], and the greater the strength of a single node, the higher the degree of centrality of that node in the network. The centrality metric is reported as a normalized value ("z score").

### Network stability

Following a previous study [39], this study tested the stability of the network analysis results by evaluating the accuracy of the edge weights and the stability of the centrality index using the bootnet (version 1.5) package. This includes the following three aspects: First, the accuracy of the edge weights was tested by calculating 95% confidence intervals using the bootstrapping method; the number of replicate samples in this study was 1000, and the test was a nonparametric test. Second, the stability of the node centrality index (in this study, strength) was tested by calculating the correlation stability (CS) coefficient using the sample reduction bootstrap method. In general, CS > 0.7 is optimal, and the CS coefficient should not be lower than 0.25, preferably higher than 0.5. Finally, in order to test whether there is a significant difference between node strength and edge weights, the bootstrap difference test for node strength and edge weights was also performed using the non-parametric bootstrapping method, with the same number of repetitions, 1000.

### Network comparison test

This study used the NetworkComparisonTest (version 2.2.1) package to compare network structures between gender (male/female), place of residence (urban/rural), and the presence of somatic chronic comorbidity (lack of chronic comorbidit/presence of somatic chronic comorbidity). This technique for direct comparison between different network structures is known as the network comparison test (NCT).NCT uses a permutation test where different two sets of network structures are repeated 1000 times.NCT was used to test for invariance between network structures [40] and the results consisted of three aspects: (i) invariance of network structures (ii)invariance of overall strength, and (iii)invariance of borderline strength.

## Results

### Study sample

A total of 501 community-dwelling older adults completed the survey and met the inclusion criteria for this study. Among them, 50.1% were male and 49.9% were female; 52.69% lived in urban areas and 47.31% in rural areas; 51.9% had no somatic chronic comorbidities while 48.1% had physical chronic comorbidities. The mean age of the respondents was 71.79 years (SD = 7.14), and the means of the total scores of GAD-7, PHQ-9 and PMS were 2.10 (SD = 3.07), 3.18 (SD = 4.02), and 26.80 (SD = 6.08), respectively (Table 1). The prevalence of anxiety was 18.96%, that of depression was 26.95%, and that of combined anxiety–depression was 14.97%. Table 2 shows the mean scores, standard deviations, strength values, bridge strength values, and predictable values for each symptom of PMS, PHQ-9, and GAD-7.

**Table 1:** Demographic characteristics of the study sample (*n* = 1057)

Variables	
Age, mean (SD)	71.79 (7.14)
Female gender, n (%)	250 (49.9%)
Rural residence, n (%)	237 (47.3%)
Somatic chronic comorbidities, n (%)	241 (48.1%)
GAD-7 total, mean (SD)	2.10 (3.07)
PHQ-9 total, mean (SD)	3.18 (4.02)
PMS total, mean (SD)	26.80 (6.08)

*PHQ-9 *Nine-item Patient Health Questionnaire, *GAD-7* Seven-item Generalized Anxiety Disorder scale, *PMS* Personal Mastery Scale

**Table 2:** Mean scores, standard deviations,strength, bridge strength and predictability for each symptom of the PMS, PHQ-9 and GAD-7

Variables	symptoms	M	SD	S	BS	Pre
Personal mastery symptoms(PMS)	PMS1:Helplessness	3.96	1.12	0.22	0.32	0.64
	PMS2:Inability to change	3.94	1.17	1.01	0.13	0.73
	PMS3:Involuntarily	4.24	1.03	0.95	0.43	0.65
	PMS4:Uncontrollability	4.12	1.11	0.89	0.17	0.68
	PMS5:Inextricability	4.06	1.17	-0.77	0.18	0.49
	PMS6:Can do anything	3.03	1.37	0.72	0.23	0.69
	PMS7:Depend on oneself	3.47	1.27	-0.50	0.09	0.63
Anxiety symptoms (GAD-7)	GAD1:Nervousness	0.38	0.65	1.38	0.48	0.58
	GAD2:Uncontrollable worry	0.38	0.63	0.57	0.43	0.62
	GAD3: Worry too much	0.28	0.56	-0.01	0.32	0.60
	GAD4:Trouble relaxing	0.24	0.52	0.23	0.55	0.58
	GAD5:Restlessness	0.17	0.43	-0.21	0.42	0.55
	GAD6: Irritability	0.40	0.64	0.57	0.74	0.56
	GAD7:Feling afraid	0.26	0.52	-0.43	0.25	0.49
Depression symptoms (PHQ-9)	PHQ1:Anhedonia	0.45	0.66	-0.46	0.35	0.49
	PHQ2:Sad mood	0.36	0.65	1.22	0.65	0.65
	PHQ3:Sleep	0.66	0.97	-1.12	0.35	0.38
	PHQ4:Energy	0.59	0.78	-0.13	0.21	0.52
	PHQ5:Appetite	0.28	0.58	-2.02	0.25	0.25
	PHQ6:Guilty	0.21	0.53	0.76	0.41	0.57
	PHQ7:Concentration	0.19	0.49	-0.98	0.29	0.49
	PHQ8:Motor	0.35	0.71	0.57	0.27	0.51
	PHQ9:Suicide	0.07	0.32	-2.47	0.19	0.31

*M* mean, *SD* Standard deviation, *S* Strength, *BS* Bridge Strength, *Pre* Predictability

### Network structure and centrality measure analysis

**Fig. 1:**
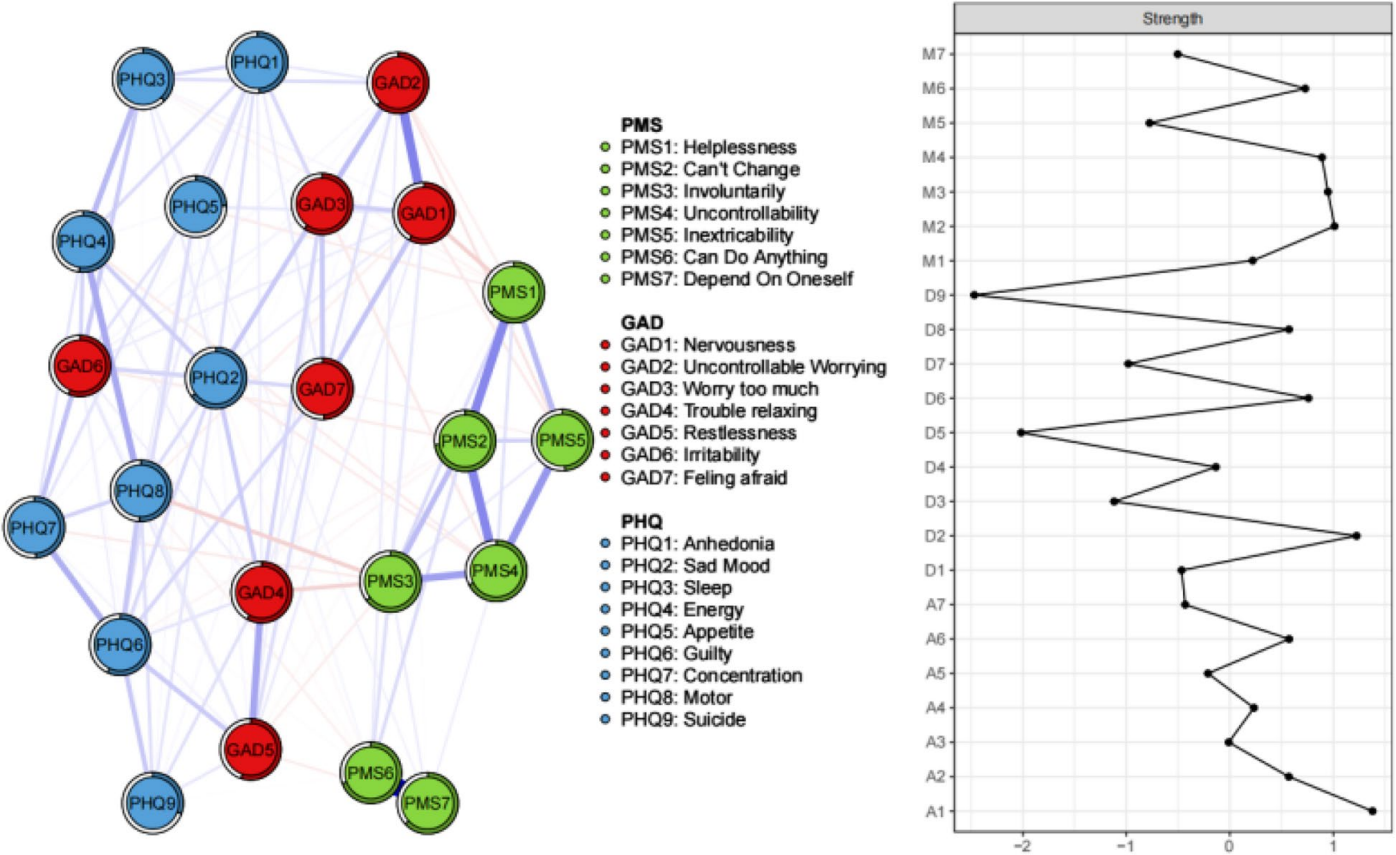
Symptom network model of anxiety-depression-individual mastery in community-dwelling older adults. The peripheral ring pie chart of each node represents predictability (the greater the proportion of the ring being darker, the more predictable the node is). The red nodes represent symptoms of anxiety (GAD-7), the blue nodes represent symptoms of depression (PHQ-9), and the green nodes represent symptoms of personal mastery (PMS). The blue line between nodes indicates positive correlation, red indicates negative correlation, and the width of the edge line represents the closeness of the relationship between the nodes

The network of anxiety–depression–personal mastery was analyzed using the EBICglasso model (Fig. 1). The predictability of symptoms is presented in Fig. 1 as a circular pie chart. The average predictability for community-dwelling older adults was 0.55. The network analysis plot of anxiety–depression–personal mastery among older adults in the community shows that node PMS6 ("Can do anything") and node PMS7 ("Depend on oneself") are most closely associated (edge weight = 0.69), followed by the association between node GAD1 ("nervousness") and node GAD2 ("Uncontrollable worry"), (edge weight = 0.33); the association between node PMS2 ("inability to change") and node PMS4 ("no control"), (edge weight = 0.32) and the association between node PMS2 ("inability to change") and link between node PMS1 ("helpless") (edge weight = 0.31).

On the central metric of this study, strength, node GAD1 ("Nervousness", strength = 1.38) had the highest strength, followed by PHQ2 ("Sad mood", strength = 1.22), PMS2 ("Inability to change", strength = 1.01) and PMS3 ("Involuntarily", strength = 0.95) (Fig. 1). The above four symptoms scored higher on the indicator of strength than the other symptoms in the network and can be considered as central symptoms in the symptom network of anxiety–depression–personal mastery.

### Bridge symptoms and bridge connections

**Fig. 2:**
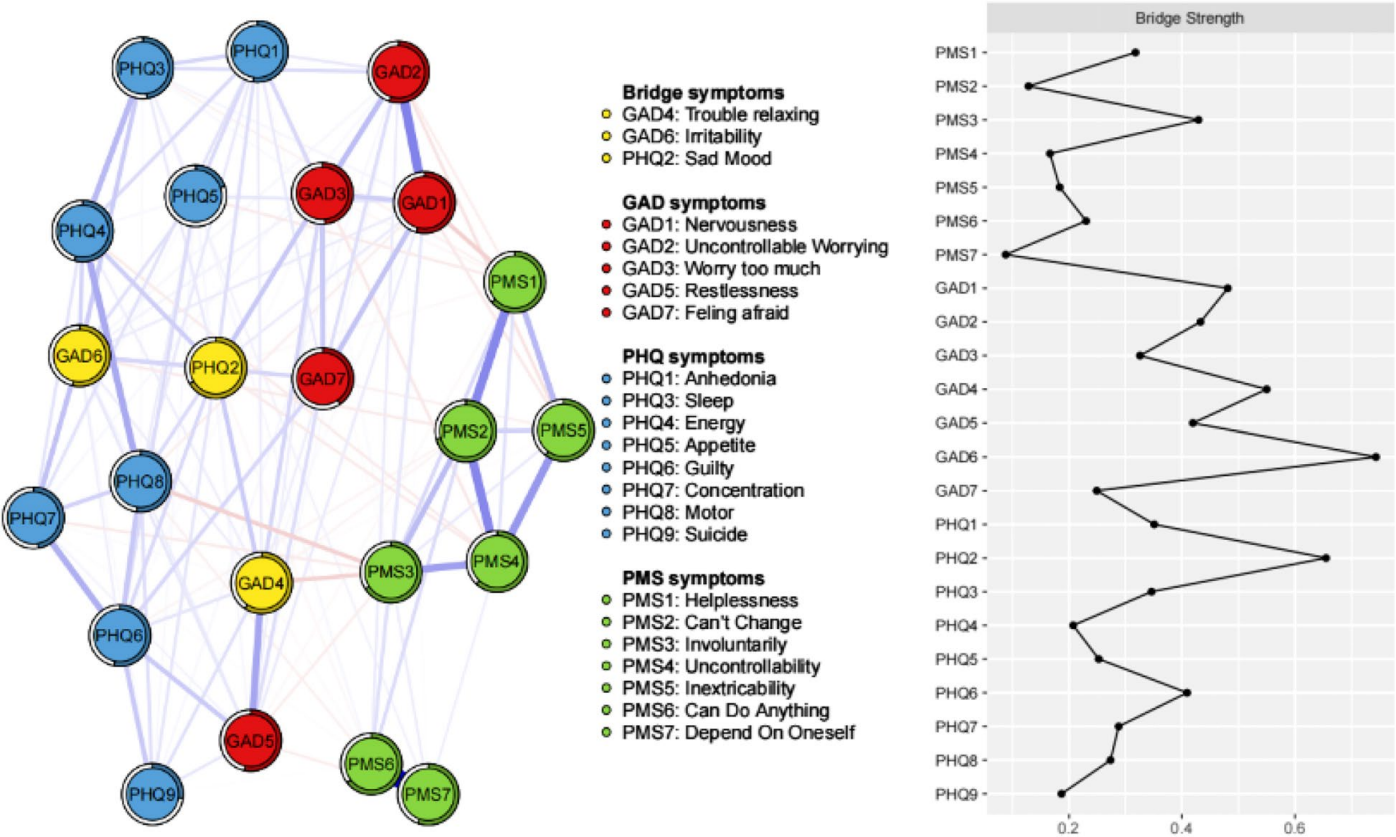
The network structure of Anxiety-Depression-Personal Mastery in community-dwelling older adults using yellow marker bridge symptoms. The peripheral ring pie chart of each node represents predictability (the greater the proportion of the ring being darker, the more predictable the node is). The red nodes represent symptoms of anxiety (GAD-7), the blue nodes represent symptoms of depression (PHQ-9), and the green nodes represent symptoms of personal mastery (PMS). The blue line between nodes indicates positive correlation, red indicates negative correlation, and the width of the edge line represents the closeness of the relationship between the nodes

In this symptom network, the main bridge symptoms were nodes GAD6 ("Irritability"**,** bridge strength = 0.743), PHQ2 ("Sad mood", bridge strength = 0.655), and GAD4 ("Trouble relaxing", bridge strength = 0.550). Moreover, the three main pathways of bridge connectivity were "Irritability" and "Concentration" (GAD6-PHQ7, egde weight = 0.159), "Irritability" and "Inability to change" (GAD6- PMS2, egde weight = -0.055), and "Sad mood" and "Uncontrollability" (PHQ2-PMS4, egde weight = -0.053) (Fig. 2).

### Network accuracy and stability

**Fig. 3:**
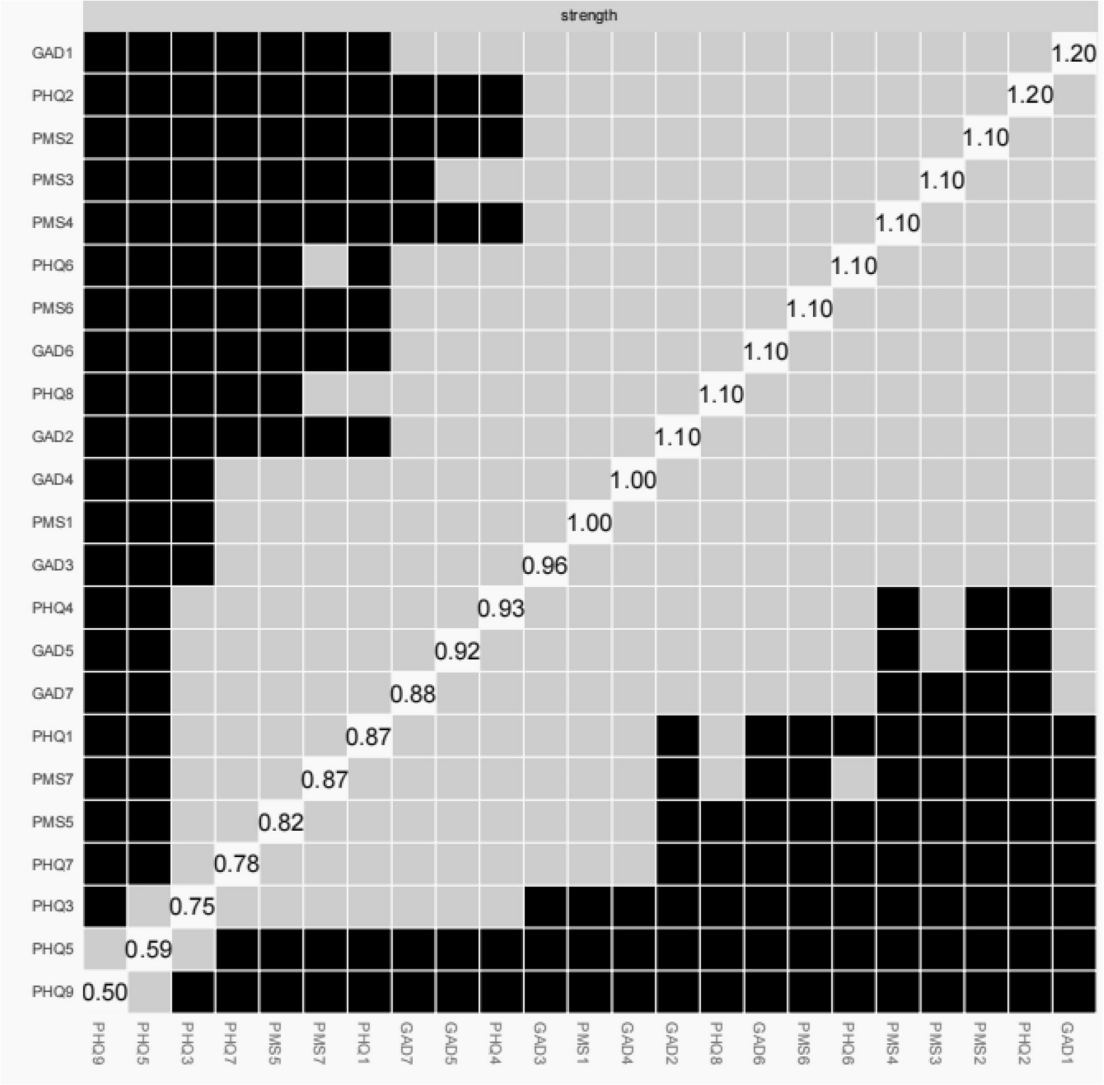
Nonparametric bootstrap difference test for strength. Gray boxes indicate non-significant differences between nodes, while black boxes indicate significant differences (α = 0 .05). The values reported in the diagonal line indicate the strength values of the nodes

First, bootstrap 95% confidence interval estimates of the edge weights via nonparametric estimation showed stable results for the edge weights (Figure S1). Second, for the stability test of the nodes in the centrality metric, the stability level of the strength was good (CS coefficient = 0.673), implying that a maximum of 67% sample reduction could be accepted without a significant impact on the network (Figure S2). Finally, for the tests of edge differences, the results of the bootstrap difference tests for edge weights showed that most of the comparisons between edge weights were statistically significant and that the edge weights were stable (Figure S3); the results of bootstrap difference tests for strength showed that nodes GAD1 ("Nervousness"), PHQ2 ("Sad mood"), PMS2 ("Inability to change"), and PMS3 ("Involuntarily") had more significant differences and were more central than other nodes in the network (Fig. 3). In contrast, some other nodes had less severe symptoms, such as PHQ3 ("Sleep"), PHQ5 ("Appetite"), and PHQ9 ("Suicide").

### Network comparison tests

In this study, network comparison tests were conducted separately between gender (male/female), place of residence (urban/rural), and presence of somatic chronic comorbidity (no somatic chronic co-morbidity/somatic chronic co-morbidity). (1) There were no significant differences in network structure (M = 0.24, *p* = 0.394) or global strength (global strength = 10.88 for male older adults and global strength = 10.77 for female older adults in the community, S = 0.11, *p* = 0.79) in the network comparison test between male and female community-dwelling older adults.(2) There was no significant difference in network structure (M = 0.20, *p* = 0.794) or global strength between older adults in urban and rural communities (global strength = 10.17 for older adults in urban communities and 10.90 for older adults in rural communities, S = 0.73, *p* = 0.08) (3) There was no significant difference in network structure (M = 0.24, *p* = 0.367) or global strength (global strength = 10.17) for older adults in urban communities and 10.90 for older adults in rural communities, S = 0.73, *p* = 0.08).Between older adults with no somatic chronic comorbidity and those with chronic co-morbidities, there were also no significant differences in network structure(M = 0.24, *p* = 0.367) or global strength (global strength for older adults with non-somatic chronic comorbidity = 10.53, global strength for older adults with somatic chronic comorbidity = 11.07, S = 0.53, *p* = 0.21).

## Discussion

### The symptoms of anxiety and depression are closely related, with the symptom nodes of personal mastery negatively correlated with the symptom nodes of anxiety and depression

The results of the present investigation show that the prevalence of anxiety was 18.96%, the prevalence of depression was 26.95%, and the prevalence of combined anxiety and depression was 14.97%. These figures are higher than previous studies in community-dwelling older adults, which reported rates of 9.9% for anxiety and 25.5% for depression [1, 2]. Notably, in economically developed areas like the Jiangnan region, anxiety and depression were more prevalent. Future research should explore variations in less developed regions to understand the correlation between economic development and psychological disorders in elderly communities. Furthermore, comorbid anxiety and depression are prevalent, highlighting their strong correlation. Early conceptions of depression emphasized the central role of anxiety [41]. For instance, in the DSM-5, the depressive response, a precursor to Major Depressive Disorder (MDD), is characterized as a form of subdued anxiety [42]. Diagnoses of anxiety and depression frequently coincide, with highly correlated symptoms. They act as mutual risk factors, and experiencing one substantially increases the likelihood of developing the other, resulting in comorbid anxiety and depression [3].

Personal mastery, a vital psychological resource, tends to follow an "inverted U-shape" pattern with age [43], meaning it decreases as individuals get older. In this study, community-dwelling elderly individuals had a moderate personal mastery score of 26.80 ± 6.08, which was in the middle level and had room for improvement, aligning with previous research [44]. Importantly, each symptom in the personal mastery dimension was negatively correlated with anxiety and depression symptoms. From a positive psychology perspective [24], personal mastery is an important psychological resource. Individuals with higher levels of personal mastery are more confident in life. They tend to be able to face various events in their lives with ease, whether they are unexpected or negative. As a result, they often maintain a sense of relief rather than being overly tense all the time because they believe that everything is under control. Individuals with higher levels of personal mastery have correspondingly more stable emotions. They also have negative emotions, but individuals with a high level of personal mastery are able to digest these emotions well in most cases.In other words, individuals with high levels of personal mastery have more positive mentalities and fewer negative emotions, and the vulnerability and susceptibility to anxiety and depression is greatly reduced [45]. The importance of personal mastery to an individual's mental health cannot be overstated. It was found [44] that among older adults in the community, positive and negative emotion scores and family caring were influential factors for personal mastery, suggesting that we should encourage older adults to express positive emotions, reduce negative emotions, and increase family caring. Additionally, interventions like cognitive emotion management and attribution therapy [20] offer strategies to bolster personal mastery in community-dwelling older adults.

### GAD1、PHQ2、PMS2 and PMS3 are the central symptoms in the anxiety-depression personal mastery network of community-dwelling older adults

"Nervousness" (GAD1) is a central symptom in the anxiety-depression-personal mastery network among community-dwelling older adults. In Fig. 1, it's evident that "Nervousness" (GAD1) and "Uncontrollable worry" (GAD2) are closely connected. "Nervousness" represents uneasiness in response to impending disasters, while "Uncontrollable worry" involves recurring concerns about undesirable future events [46]. "Nervousness" typically relates to short-term events, while "Uncontrollable worry" pertains to long-term events [47]. This strong association between "Nervousness" (GAD1) and "Uncontrollable worry" (GAD2) aligns with previous research [48]. In a study [49] of the British population, which included a network analysis of anxiety and depression, the most central symptom was "Trouble realxing" (GAD4), a result that differs from the present study. The differences in findings may be due to cultural differences. Certainly, there is limited research based on a network analysis approach to explore anxiety and depression in the British population. Therefore, this still needs to be verified in the future studies. Whereas in China, due to the influence of Chinese Confucianism; older adults in traditional Chinese families play the role of leaders and elders [48], and they like to plan ahead. Whether dealing with a crisis or a risk that may occur in the long or short term, traditional Chinese older adults want to be able to deal with problems in advance, so they unconsciously worry too much, and "Nervousness" and "Uncontrollable worry" are born together.

However, "Nervousness" (GAD1) was the central symptom in the anxiety–depression–personal master symptom network in community-dwelling older adults, which is different from previous studies. A previous study [38] of adolescents showed that "Sad mood" (PHQ2) is the most central node in a network model of anxiety and depression. This difference is likely due to the fact that the two are vastly different in age and at different stages of life. Adolescents are more vulnerable, they are less receptive to negative events in their growth process and are more prone to sadness [38]; whereas older adults have more experience and are better at dealing with various emergencies in their lives. However, due to the need to worry about their children and families, older people tend to become nervous easily [48]. Another previous studie [30] found that "Uncontrollable worry" (GAD2) was the node with the highest central value in anxiety-depression networks. This reflects the distinction between community-dwelling older adults and those with functional impairment who cannot independently perform daily activities. Community-dwelling older adults can better care for themselves, resulting in less concern about distant future events compared to their functionally impaired counterparts. Additionally, another study [50] of pre-pandemic Chinese older adults also demonstrated that "Uncontrollable worry" (GAD2) was the most central symptom of the anxiety dimension in the network model. The COVID-19 pandemic shifted the mindset of community-dwelling older adults [51], focusing them on immediate life events and a desire for better daily living [48]. This shift explains why "Nervousness" is now the central symptom of anxiety and depression in this population.

"Sad mood" (PHQ2) is a central symptom in the anxiety-depression-personal mastery network among community-dwelling older adults, consistent with previous research on anxiety-depression symptom networks in functionally impaired older adults [30]. In addition, "Sad mood" (PHQ2) is also the most central symptom in inpatients [52] and psychiatric patients receiving treatment for mood, anxiety, personality, and psychotic disorders [53].The fact that "Sad mood" (PHQ2) was the predominant core symptom in all network models of anxiety and depression across a variety of populations certainly emphasizes the importance of sadness and its commonality across the majority of the population.This reaffirms its importance in diagnosing major depression according to the DSM-5 criteria [54]. "Sad mood" has long been recognized as a key manifestation of depression, and its centrality in this network structure highlights its role as a predictor not only for depression but also for anxiety. As Chinese older adults age, declining physical functions pose self-care challenges. This fosters a heightened awareness of their aging, reduced economic and social status, and a growing sense of worthlessness. They often view themselves as burdens to their families, leading to negative attitudes towards aging. Additionally, the presence of acute or chronic illnesses and stressful events like the loss of loved ones intensify negative emotions, compounded by a constant exposure to death-related information [55]. Moreover, it's essential to note the lack of death education among older adults in the Chinese community [56]. Traditional cultural norms hinder open expression of grief when faced with loss, leading to emotional suppression [57] and avoidance of death-related discussions [56]. Addressing this issue, government and community initiatives should prioritize death education for community-dwelling older adults to foster a healthier perspective on mortality. Additionally, the strong connection between "Nervousness" (GAD1) and "Sad mood" (PHQ2) is noteworthy, indicated by a thick edge with high predictability. This indicates a robust association between these two symptoms, with a positive influence on each other (indicated by a blue edge). Thus, addressing "Sad mood" among community-dwelling elderly individuals can not only reduce depression but also alleviate "Nervousness" and anxiety concurrently. This finding supports previous research indicating a close relationship between anxiety and depressive symptoms, with mutual influence [58].

### The symptom "Inability to change" (PMS2) is a central element in the anxiety-depression

-personal mastery network among community-dwelling older adults. This study, notably, is the first to explore all seven personal mastery symptoms within this network. This finding is deeply rooted in traditional Chinese culture, where older adults often adopt a fatalistic perspective, believing that elements like economic status and health are predetermined. Consequently, they may lack confidence and motivation to change. When faced with negative life events, transitions, or declining health, this sense of "Inability to change" becomes more pronounced, elevating the risk of anxiety and depression. The absence of personal mastery, combined with the belief that many critical life aspects are beyond their control, can disrupt emotional management and psychological regulation in older adults living in the community. Thus, the "Inability to change" symptom significantly affects other personal mastery symptoms and contributes to anxiety and depression. The occurrence of "Inability to change" may cause individuals to lose their motivation to manage and regulate their emotions, resulting in a loss of motivation and initiative to face difficulties in the community. Accumulated unresolved negative emotions further foster anxiety and depression. Addressing this symptom requires targeted interventions, such as attribution training [20]. Additionally, government, community managers, and healthcare workers can enhance social support through regular visits, provide more social activities, promote regular physical exercise, and offer improved health education for self-care among older adults. These small adjustments can help community-dwelling seniors shift negative attitudes, bolster their sense of control over their lives, and reduce anxiety and depression by reigniting their self-motivation.

It's important to highlight that "Involuntarily" (PMS3) is another central symptom in this study's symptom network. Moreover, the edge weights between "Involuntarily" (PMS3) and "Inability to change" (PMS2) nodes were notably high, denoting a very close connection between them. Both nodes also demonstrated very high predictability, especially "Inability to change" (PMS2), signifying that "Involuntarily" (PMS3) and "Inability to change" (PMS2) mutually influence each other, with "Inability to change" being particularly susceptible to the influence of "Involuntarily." Furthermore, the edge weight between "Involuntarily" (PMS3) and the previously central symptom "Nervousness" (GAD1) was also substantial, indicating a strong association between them. Considering these interconnections among central symptoms, it's evident that "Involuntarily" (PMS3), despite having the lowest centrality score among the four central symptoms, plays a crucial role in the network. This underscores the importance of giving focused attention to "Involuntarily" (PMS3) when conducting further research or developing precise intervention programs.

### GAD6、PHQ2 and GAD4 are the bridge symptoms in the anxiety-depression-personal mastery network of community-dwelling older adults

In a symptom network, symptoms can influence each other, potentially leading to the development of other mental disorders. Those symptoms that increase the risk of developing other mental disorders are called bridge symptoms [59, 60]. Studies have shown that bridge strength is the best indicator for identifying bridge symptoms. Bridge strength refers to the total connectivity of a node with other disorders. In this study, "Irritability" (GAD6), "Sad mood" (PHQ2), and "Trouble relaxing" (GAD4) had the highest bridge strength values, serving as bridges connecting anxiety, depression, and personal mastery symptoms. This is generally consistent with previous findings [61], where the bridge symptoms analyzed in the depression and anxiety network of Macau residents during the COVID-19 outbreak also included "Irritability" (GAD6) and "Sad mood" (PHQ2).

As can be seen in Fig. 2, there is a strong association between the bridge symptoms "Sad mood" (PHQ2) and "Irritability" (GAD6) and between "Sad mood" (PHQ2) and "Trouble relaxing" (GAD4). These three key bridge symptoms are intricately interconnected and mutually influential. Furthermore, "Sad mood" (PHQ2) emerges as both a central symptom and a pivotal bridge symptom tightly linked to others, underscoring its significance in the overall network structure.This finding is consistent with previous research that nodes of "Sad mood" (PHQ2) are important in both single depression networks [29], anxiety–depression networks [53], and the anxiety–depression–personal masternetwork structure constructed in this study. For this, it is possible to focus on the causes and influencing factors of "Sad mood" symptoms in the development of precise interventions. Similarly, "Irritability" (GAD6) and "Trouble relaxing" (GAD4) act as bridge symptoms that mediate the entire network.

The primary bridge connections involve GAD6 ("Irritability")–PHQ7 ("Difficulty focusing"), GAD6 ("Irritability")–PMS2 ("Inability to change"), and PHQ2 ("Sad mood")–PMS4 ("Inability to control"). If we cut these bridge symptoms, we can block the pathway of activation from one mental disorder to another. This means that individuals won't progress from a single mental disorder to experiencing multiple mental disorders together. Therefore, by identifying "Irritability" (GAD 6) and "Trouble relaxing" (GAD 4) as potential causes and intervening to address them, we can effectively prevent complications. This way, psychological disorders can remain isolated as either anxiety, depression, or low personal mastery disorders, making them more amenable to targeted interventions.

### In NCT, network analysis of different residence, somatic chronic comorbidities and gender had no significant effect in community-dwelling older adults

A previous study [28] regarding the network analysis of anxiety and depression among older Korean adults found significant differences in global strength and network structure by gender. For this reason, in the present study we conducted an NCT on the collected data but found no significant differences in network analysis by gender. The study showed [62] that older community-dwelling adults with somatic chronic comorbidities had significantly higher anxiety and depression problems than those without somatic chronic comorbidities. In the present study, we also performed NCT for the presence or absence of somatic chronic co-morbidity and found no significant differences in network analysis for the presence or absence of somatic chronic co-morbidity. Additionally, in this study, we conducted NCTs of urban and rural community-dwelling elders and found no differences in the network structure between urban and rural areas either.

No differences in all three comparisons suggests that the network structure of anxiety, depression, and personal mastery in community-dwelling older adults is not influenced by gender, somatic chronic comorbidities, or urban–rural geography. This also implies that there are universal and common qualities of psychological disorders that require universal attention and screening of all community-dwelling older adults for symptoms of these psychological disorders.

## Limitations

The limitations of this study are as follows:

- First, the sample size included in this study is still small and the scope of the survey is not broad enough. In this regard, a cross-sectional survey with more samples and a broader scope of investigation can be performed in the follow-up study.
- Second, since this study is only a cross-sectional survey, it is not possible to derive the true causal relationship between symptoms, instead providing a basis for subsequent research on the longitudinal dynamic network structure.
- Third, the Cronbach′s α coefficient for personal mastery in this study was slightly lower than in previous studies, which may be due to the heterogeneity of the survey population. Nonetheless, the Cronbach′s α coefficient of personal mastery in the present study was still higher than the minimum requirement of Cronbach′s α coefficient.
- Fourth, this survey was conducted on community-dwelling older adults in China, and the generalization of the findings obtained from this study is limited due to the variability among different countries, cultures, and ethnic groups. Moreover, it is prudent to note that data were only collected from community-dwelling older adults in more economically developed Jiangnan regions of China, and no data were collected from community older adults in less economically developed regions of China. Therefore, it was not possible to compare the symptom structure of psychological disorders between older adults in communities in economically developed and less-developed regions of China. It is hoped that further comparisons can be made in subsequent studies, which in turn will provide a basis for developing more specific intervention programs for community-dwelling older adults in regions with different economic statuses in China.

## Conclusion

In conclusion, this study addresses the anxiety–depression network analysis of community-dwelling older adults and newly incorporates the personal mastery dimension, seeking to provide new network analysis perspectives on aspects of psychological disorders in community-dwelling older adults. This study identifies for the first time the central symptoms (nervousness, sad mood, inability to change, and involuntarily) and bridge symptoms (irritability, sad mood, and trouble relaxing) of anxiety, depression, and personal mastery network structure in community-dwelling older adults and provides a scientific basis for the psychological disorders of community-dwelling older adult. This provides a scientific basis for the development of precise intervention programs for the prevention and treatment of psychological disorders in the community-dwelling elderly. Finally, it is also noteworthy that half of the nodes in the central symptoms (inability to change, involuntarily) come from the personal mastery dimension, which reflects the key role of personal mastery in this network of psychological disorders.

### Supplementary Information

**Supplementary Material 1.**

## Data Availability

The datasets used and/or analyzed in this study are available from the corresponding authors upon reasonable request.

## Abbreviations

PHQ-9: nine-item Patient Health Questionnaire
GAD-7: seven-item Generalized Anxiety Disorder scale
PMS: Personal Mastery Scale
LASSO: Least Absolute Shrinkage and Selection Operator
M: mean
SD: Standard deviation
S: Strength
BS: Bridge Strength
Pre: Predictability
MDD: Major Depressive Disorder
NCT: Network Comparison Test
EBIC: Extended Bayesian Information Criterion
CS: correlation stability
DSM: Diagnostic and Statistical Manual of Mental Disorders
