# Supplementary material for: Associations between anxiety, depression, and personal mastery in community-dwelling older adults: a network-based analysis
Source: BMC Psychiatry. 2024 Mar 7;24:192. doi: 10.1186/s12888-024-05644-z (PMC10921593; doi:10.1186/s12888-024-05644-z)
Supplement: Supplementary file 1 — Supplementary Material 1. [file 12888_2024_5644_MOESM1_ESM.docx]

**Supplementary Material**

Figure S1. Bootstrap 95% confidence intervals for nonparametric estimation of edge weights

Figure S2 Stability test of nodes on the centrality metric

Figure S3 Bootstrap difference test for edge weights.


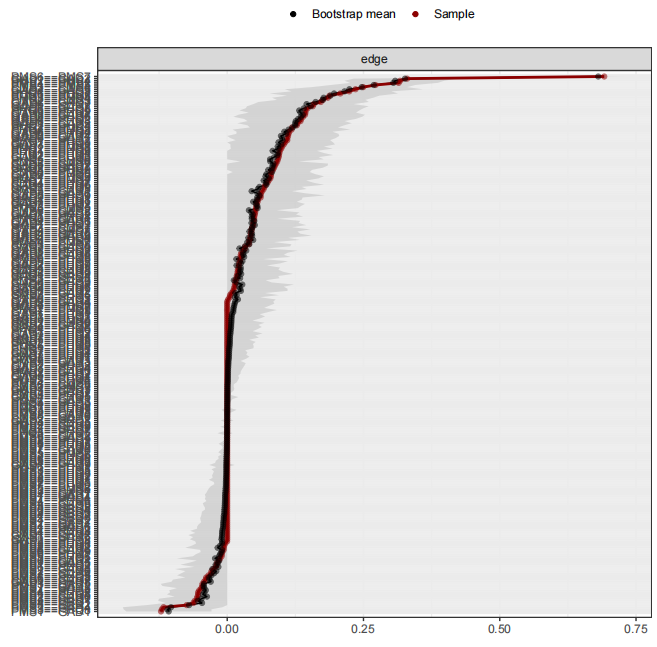


Figure S1 Bootstrap 95% confidence intervals for nonparametric estimation of edge weights. The black dots indicate the value of each edge weights, ordered from the highest to the lowest value. The gray area indicates the 95% confidence interval of the edge weights, estimated using a nonparametric bootstrap procedure (bootstrap net package). Wide intervals indicate low stability and narrow intervals indicate high stability.


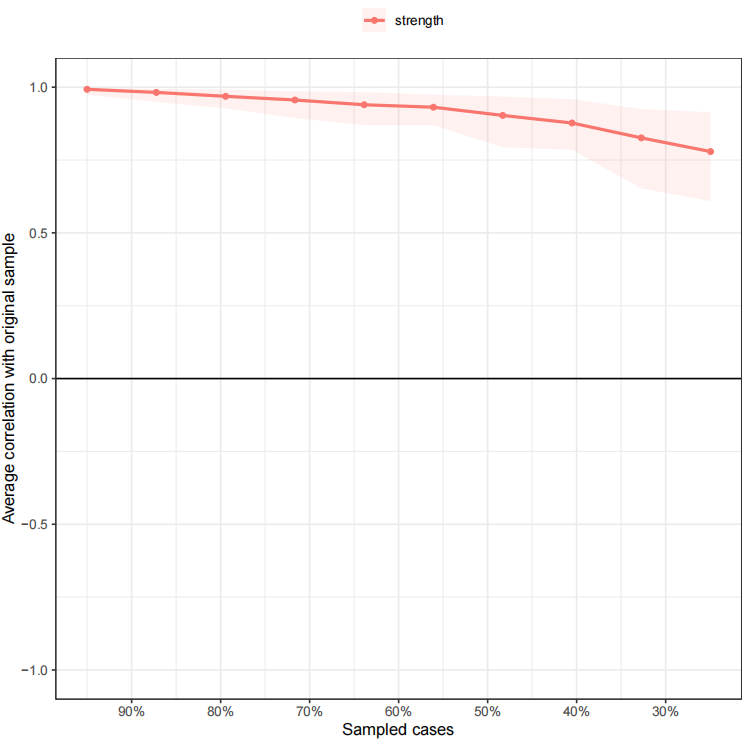


Figure S2 Stability test of nodes on the centrality metric. x-axis is the percentage of samples used at each step, and y-axis is the degree of association between the original sample and the estimated results from the sample reduction. The red line indicates the specific value of the node in emphasizing centrality.


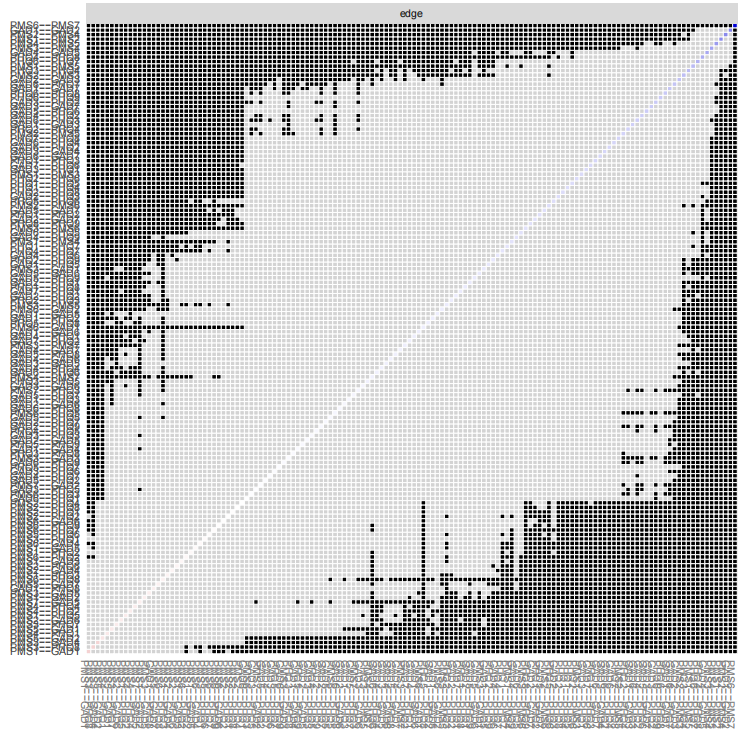


Figure S3 Bootstrap difference test for edge weights. Gray boxes indicate no significant differences between edges. Black boxes indicate significant differences between edges (α = 0.05). Blue boxes in the edge weights plot indicate positive correlations.
